# Mechanisms Underlying Cancer Growth and Apoptosis by DEK Overexpression in Colorectal Cancer

**DOI:** 10.1371/journal.pone.0111260

**Published:** 2014-10-23

**Authors:** Lijuan Lin, Junjie Piao, Yibing Ma, Tiefeng Jin, Chengshi Quan, Jienan Kong, Yulin Li, Zhenhua Lin

**Affiliations:** 1 Department of Pathology & Cancer Research Center, Yanbian University Medical College, Yanji, China; 2 Department of Medical Imaging, College of Medicine, Eastern Liaoning University, Dandong, China; 3 Department of Pathology, Dandong Centre Hospital, Dandong, China; 4 The Key Laboratory of Pathobiology, Ministry of Education, Bethune Medical College, Jilin University, Changchun, China; University of Kentucky College of Medicine, United States of America

## Abstract

Our previous study indicated that DEK protein was overexpressed in colorectal carcinoma (CRC) compared with the normal colorectal mucosa. DEK was also significantly correlated with the prognostic characteristics of patients with CRC, demonstrating that DEK played an important role in CRC progression. In this work, we evaluate the effects of DEK on biological behaviors in CRC and explore the related molecular mechanisms. The results showed that DEK was overexpressed in human CRC tissues, and was correlated with the Ki-67 index and the apoptotic index. DEK depletion by RNAi in SW-620 and HCT116 cells significantly decreased cell proliferation, but increased cell apoptosis. Upregulation of DEK was involved in the p53/MDM, Bcl-2 family, and caspase pathways. Our study demonstrates that DEK promotes the growth of CRC, and could be a therapeutic target in CRC.

## Introduction

Colorectal carcinoma (CRC) is the third most common malignancy and the second most common cause of cancer death worldwide [1]. The quality of life and the 5-year survival rate are low in advanced CRCs even with surgical excision accompanied by chemotherapy and radiotherapy. Early detection of CRC is crucial for successful treatment because advanced high-grade disease is correlated with increased metastasis and mortality [2]–[4]. Therefore, the identification of new CRC mediators and biomarkers, particularly those associated with metastasis and growth, remains critical to combat mortality from recurrent disease. The progress in cancer pathogenesis can help unravel the vital/valid molecular biomarkers involved in colorectal carcinogenesis and assist in developing and discovering novel therapeutic interventions, and preventive strategies and agents. Our previous data have shown that DEK protein expression is upregulated in CRC tissues [5]. The overexpression is particularly marked in high-grade and late-stage CRCs, making DEK a potential new biomarker for the prognosis of CRC and a target in the fight against recurrence [5].

DEK was originally discovered as the target of a chromosomal translocation event t(6;9) in a subset of acute myeloid leukemias [6]–[7]. Now, DEK is emerging as a member of a novel class of DNA topology modulators that can be targets and effectors of pro-tumorigenic events [8]. DEK locates at chromosome 6p22.3 [9], and is a highly conserved nucleoprotein that can be phosphorylated. Composed of 375 amino acids, DEK is mainly distributed in the nucleus euchromatin, and preferentially expresses in actively proliferating and malignant cells, where it can reach up to 4 to 6 million copies per nucleus [8]. Subsequent studies have repeatedly identified DEK as a frequently overexpressed gene in a number of neoplasms [10]–[12]. Furthermore, DEK can exert effects on mRNA splicing, transcriptional control, DNA damage repair, differentiation, cell viability and cell-to-cell signaling [11], [13]–[15]. However, the functions of DEK *in vitro* in CRC cellular behavior have not been evaluated. Previously, we showed that DEK protein was overexpressed in 109 cases of CRC tissues, was significantly correlated to the patients’ prognosis characteristics, and was an independent risk factor for overall survival [5]. This study observes the expression of DEK in a new group of collected primary CRCs, and correlates DEK expression with the Ki-67 and apoptotic indices, which reflect the contributions of cell proliferation and cell loss, respectively. Also, we clarify the role of DEK in CRC progression with DEK RNA interference (RNAi) in a cell line derived from a CRC.

DEK is an inhibitor of p53-dependent and -independent cellular senescence and apoptotic phenotypes [7], [14], [16], and transcriptionally upregulated by the Rb/E2F pathway, which is frequently perturbed in CRC [17]–[19]. Therefore, its expression is strongly indicative of proliferation and apoptosis. Here we define specific oncogenic activities of DEK in CRCs *in vitro*, and identify a molecular mechanism through which DEK contributes to tumor growth.

## Methods

### Ethic Statement

All participants gave written informed consent for the study that complied with the Helsinki Declaration and was approved by the Human Ethics and Research Ethics committees of Yanbian University Medical College in China. Through the surgery consent form, all the participants were informed that the resected specimens were stored by the hospital and potentially used for scientific research, and that their privacy would be maintained.

### Tissue specimens

Fresh samples from four cases of CRC were paired with adjacent noncancerous tissues, and were included with the routinely processed and diagnosed 55 cases of colorectal cancer tissues that were selected randomly from patients undergoing surgery between 2009 and 2012 at the Tumor Tissue Bank of Yanbian University Medical College. The pathological parameters were carefully reviewed in all of the cases. A total of 22 of the adjacent normal colon mucosa tissues from the cancer resection margin and 18 of the colorectal adenoma tissues were also included. None of the patients had received chemotherapy before surgery or had distant metastases. The H&E-stained slides were reviewed by two experienced pathologists (Lin Z, Jin T) and one appropriate paraffin block was selected for this study.

### Immunohistochemical (IHC) analysis for DEK and Ki-67

The IHC staining method and interpretation criteria were as previously described [5]. Briefly, to eliminate endogenous peroxidase activity, 4 µm-thick tissue sections were deparaffinized, rehydrated and incubated with 3% H_2_O_2_ in methanol for 15 min at room temperature (RT). Antigen retrieval was performed at 95°C for 20 min by placing the slides in 0.01 M sodium citrate buffer (pH 6.0). The slides were then incubated with the DEK antibody (1∶50, BD Biosciences Pharmingen, CA, USA) and a monoclonal mouse anti-Ki-67 antibody (clone: MIB-1; Dako, Glostrup, Denmarkat 4°C overnight. After incubation with biotinylated secondary antibody at RT for 30 min, the slides were incubated with streptavidin-peroxidase complex at RT for 30 min. Immunostaining was developed using 3,3′-diaminobenzidine, and Mayer’s hematoxylin was used for counterstaining. We used mouse IgG as an isotope controls. In addition, positive tissue sections were processed while omitting of the primary antibody as negative controls.

Both DEK and Ki-67 is usually expressed in the cell nucleus. The IHC staining for DEK was semi-quantitatively scored as ‘−’ (negative, no or less than 5% positive cells), ‘+’ (5–25% positive cells), ‘++’ (26–50% positive cells) and ‘+++’ (more than 50% positive cells). Only the nuclear expression pattern was considered as positive staining, and the strongly positive means ‘++’ and ‘+++’ positive cells. The Ki-67 index (the number of Ki-67-positive tumor cells divided by the total number of tumor cells ×100%) was determined by counting the number of tumor cells in 20 randomly selected high-power fields (×400).

### Apoptotic assay in tissue sections of CRC

The apoptotic index was measured using an in situ apoptosis detection kit (Takara Bio, Otsu, Japan). The staining procedures were performed following the manufacturer’s instructions. After routine deparaffinization, the tissue section was digested with proteinase K (20 mg/mL in PBS) for 15 min at room temperature and washed with PBS. Slides were then incubated in 3% hydrogen peroxide for 5 min and washed with PBS. TdT enzyme and substrate were pipetted onto the sections, which were then incubated at 37°C for 90 min. After washing, anti-FITC HRP conjugate was added to the slides for 30 min. The slides were washed, stained with diaminobenzine (Nichirei, Tokyo, Japan), and counterstained with hematoxylin. The apoptotic index (the number of positive tumor cells divided by the total number of tumor cells ×100%) was determined by counting the number of tumor cells in 20 randomly selected high-power fields (×400) [20]. The correlations between DEK expression and the Ki-67 or apoptotic index were evaluated in above human CRC tissues.

### Western blotting

Primary antibodies to DEK (1∶1000, BD, Biosciences Pharmingen, San Diego, CA, USA), mutant-p53 (1∶2000, Santa Cruz, CA, USA), MDM2 (1∶2000, Santa Cruz, CA, USA), Bcl-2 (1∶2000, Cell Signaling, Danvers, MA, USA), Bax (1∶2000, Cell Signaling, Danvers, MA, USA), PARP (1∶2000, Cell Signaling, Danvers, MA, USA), caspase-3 (1∶2000, Cell Signaling, Danvers, MA, USA), caspase-8 (1∶2000, Cell Signaling, Danvers, MA, USA), and caspase-9 (1∶2000, Cell Signaling, Danvers, MA, USA) were used.

Fresh tissue samples of CRC were ground to powder in liquid nitrogen and lysed with SDS-PAGE sample buffer. Confluent cells were lysed in lysis buffer (50 mM Tris-HCl, pH 7.4, 150 mM sodium chloride, 0.5 mM EDTA, 0.09 units/mL aprotinin, 1 mg/mL pepstatin, 10 mM phenylmethylsulfonyl fluoride, and 1 mg/mL leupeptin). Equal protein samples (20 µg) were separated on 12% SDS polyacrylamide gels and transferred to PVDF membranes. Membranes were blocked with 5% fat-free milk in Tris-buffered saline containing 0.1% Tween-20 for 1 h at room temperature. Membranes were incubated with the primary antibody overnight at 4°C. The membrane was washed 3 times with TBST, and incubated with HRP-conjugated goat anti-mouse IgG. (Cwbiotech, Beijing, China) and HRP-conjugated goat anti-rabbit IgG (Cwbiotech, Beijing, China) at room temperature for 2 h. Then expression was detected using ECL prime western blotting detection reagent (Amersham Biosciences, Uppsala, Sweden) according to the manufacturer’s instructions. β-actin (Sigma, St. Louis, MO, USA) was used as loading control. Images were captured with the Champchemi Professional image analysis system (Sagecreation, Beijing, China), and protein bands were quantified using LANE 1D software (Sagecreation, Beijing, China).

### Reverse transcription-polymerase chain reaction (RT-PCR) and Quantitative RT-PCR (qRT-PCR)

For RT-PCR, total RNA from CRC samples and cell lines were extracted using Trizol reagent (TaKaRa, Shiga, Japan) according to the manufacturer’s instructions. First-strand cDNA was synthesized by Prime Script reverse transcriptase (TaKaRa Bio, Dalian, China) and oligo (dT) by following the manufacturer’s instructions. All PCR reactions were done in 20 µl of reaction mixture, starting with 4 min at 94°C for denaturing cDNA, then the PCR amplification was performed for 23∼36 cycles of 94°C for 15 s and 53∼58°C for 30 s to anneal the primers, and extending the primers at 72°C for 1 min. Aliquots of the PCR reaction were removed after various numbers of cycles and were resolved by electrophoresis on 3% agarose gels. Images were captured with the Champchemi Professional image analysis system (Sagecreation, Beijing, China), and quantitation was performed with LANE 1D software (Sagecreation, Beijing, China).

Real-time PCR was performed by a Bio-Rad sequence detection system according to the manufacturer’s instructions using the double-stranded DNA-specific SYBR Premix Ex TaqTM II Kit (TaKaRa, Shiga, Japan). Primer sets for specific genes are shown in Table 1. Real-time PCR reactions were done in triplicates, and threshold cycle numbers (Ct) were determined at the level that showed the best kinetic PCR parameters. No-template control was used as negative control, and melting curves were obtained to confirm specificity of the PCR product. A comparative CT method (2^−ΔΔCt^) was used to measure the relative quantification of DEK gene.

**Table 1:** List of primers used in this study.

Gene	Primer	Sequence (5′–3′)
**Primers for RT-PCR**		
DEK	forward	AAACCTAGCCAGCTTCACGA
	reverse	AGCCCCAACTCCAGAGAAAC
GAPDH	forward	GGTCTCCTCTGACTTCAACA
	reverse	ATACCAGGAAATGAGCTTGA
**siRNA Duplexes**		
DEK siRNA	sense	CGAACCAAAUGUCCUGAAA dTdT
	antisense	UUUCAGGACAUUUGGUUCG dTdT
Control siRNA	sense	UUCUCCGAACGUGUCACGU dTdT
	antisense	ACGUGACACGUUCGGAGAA dTdT

### Cell culture and transfection

Human colorectal cancer cell lines SW-620, HT29, SW-480, and HCT116 were purchased from the Cell Bank of the Chinese Academy of Medical Science (Shanghai, China), and conserved by the Cancer Research Center of Yanbian University. These cell lines were cultured in RPMI 1640 or DMEM medium (Gibco, Gaithersburg, MD, USA) supplemented with 10% fetal calf serum, 2 mmol/L L-glutamine, and 100 U/ml penicillin/streptomycin in humidified 5% CO_2_ at 37°C.

DEK siRNA (siDEK) targeted the human DEK gene, and siRNA duplexes with non-specific sequences were used as negative controls (siControl); all sequences were designed and synthesized by RiboBio (RiboBio, Guangzhou, China) (Table S1). The siDEK used in this study are shown in Table 1.

### MTT assay

A thousand cells per well were incubated in 96-well plates. Twenty-four hours later, siControl and siDEK were transfected with Lipofectamine 2000 (Invitrogen, Carlsbad, CA, USA). The siDEK sequences were added in a dose-dependent manner at 30 nM, 50 nM and 100 nM. Forty-eight hours later, 5 mg/mL MTT was added to each well. After incubation at 37°C for 4 h, the supernatants were removed carefully. Then 200 µl of dimethyl sulfoxide was added to each well and the wells were thoroughly mixed for 10 min. The absorbance value (OD) at 560 nm of each well was measured using microplate reader (TECAN-infinite M200 pro, Mannedorf, Switzerland).

### Immunofluorescence staining

The CRC cell line, SW-620, was grown on coverslips to 70% confluence, and then fixed in 4% paraformaldehyde for 10 min and permeabilized with 0.5% TritonX-100 for 10 min after 24 h. Blocking was performed with 3% bovine serum albumin fraction V (Solarbio, Beijing, China) for 1 h at room temperature. After washing with PBS, cells were incubated with mouse anti-human DEK (1∶50, BD Biosciences Pharmingen, San Diego, CA, USA) at 4°C overnight, followed by incubation with Alexa Fluor 568 goat anti-mouse IgG (H+L) (A11004, 1∶1000, Life Technologies, Carlsbad, CA, USA) for 1 h at room temperature. After washing with PBS, cells were counterstained with 49-6-diamidino-2-phenylindole (DAPI) (Beyotime, Shanghai, China) and the coverslips were mounted with Antifade Mounting Medium (Beyotime, Shanghai, China). Finally, immunofluorescence signals were visualized and recorded using Leica SP5II confocal microscope [21].

### Colony formation assay

Scrambled siDEK (SiControl) and siDEK transfected SW620 cell line were plated in 10 cm dishes at the density of 5×10^4^ cells per dish. Following day, the medium was replaced with medium containing 800–1000 µg/ml of G418 (Gibco, Gaithersburg, MD, USA). After about 7 days, medium was replaced. The assay was stopped when the colonies were clearly visible even without looking under the microscope, and it was about 2∼3 weeks of incubation at 37°C, 5% CO2. Then cells were fixed with 0.5% paraformaldehyde, stained with crystal violet and then counted according to defined size of colony [22].

### Hoechst33342 staining

Cells were plated at a density of 1×10^4^ cells/mL and were cultured for 2 to 4 days until 80% to 90% confluence. A cellular density of 0.5–3.0×10^6^ cells/mL was achieved and fixed for 10 min at room temperature with 3% paraformaldehyde (50 µl) before treatment with Hoechst 33342. Hoechst 33342 was dissolved in distilled water at 25 mg/mL and added to DMEM (dulbecco's modified eagle medium) with 2% FBS at a final concentration of 16 µg/mL for 15 min. The apoptosis rate was calculated by counting 500 cells in a fluorescent microscope.

### Flow cytometry

SW-620 cells were cultured in six-well plates, and were transfected with the siRNA-targeting DEK or the scramble siRNA for 48 h with Lipofectamine 2000 Invitrogen, Carlsbad, CA, USA. After treatment, cells were harvested by trypsin (0.25%)-EDTA and collected by centrifugation at 1000 ×g for 5 min at room temperature. Cells were washed and resuspended in binding buffer, before being labeled with Annexin V-FITC and 7AAD for 20 min. Fluorescence (DNA content) was measured by flow cytometry using standard software. Cells that were Annexin V-FITC (−) and 7AAD (−) were considered viable cells, while cells that were Annexin V-FITC (+) and 7AAD (−) were considered early-stage apoptotic cells. Cells that were Annexin V-FITC (+) and 7AAD (+) were considered late-stage apoptotic cells.

### Statistical analysis

Each experiment was performed in triplicate. All data were expressed as the mean ± SD. Statistical analysis was performed using the SPSS 17.0 statistical package (SPSS, Inc., Chicago, IL, USA), and comparisons between groups were conducted using the Student’s t-test. The correlations of the DEK expression levels with CRC and histological factors were analyzed using Fisher’s exact test. Differences were considered statistically significant at *P*<0.05.

## Results

### DEK protein is overexpressed in CRCs

**Figure 1:**
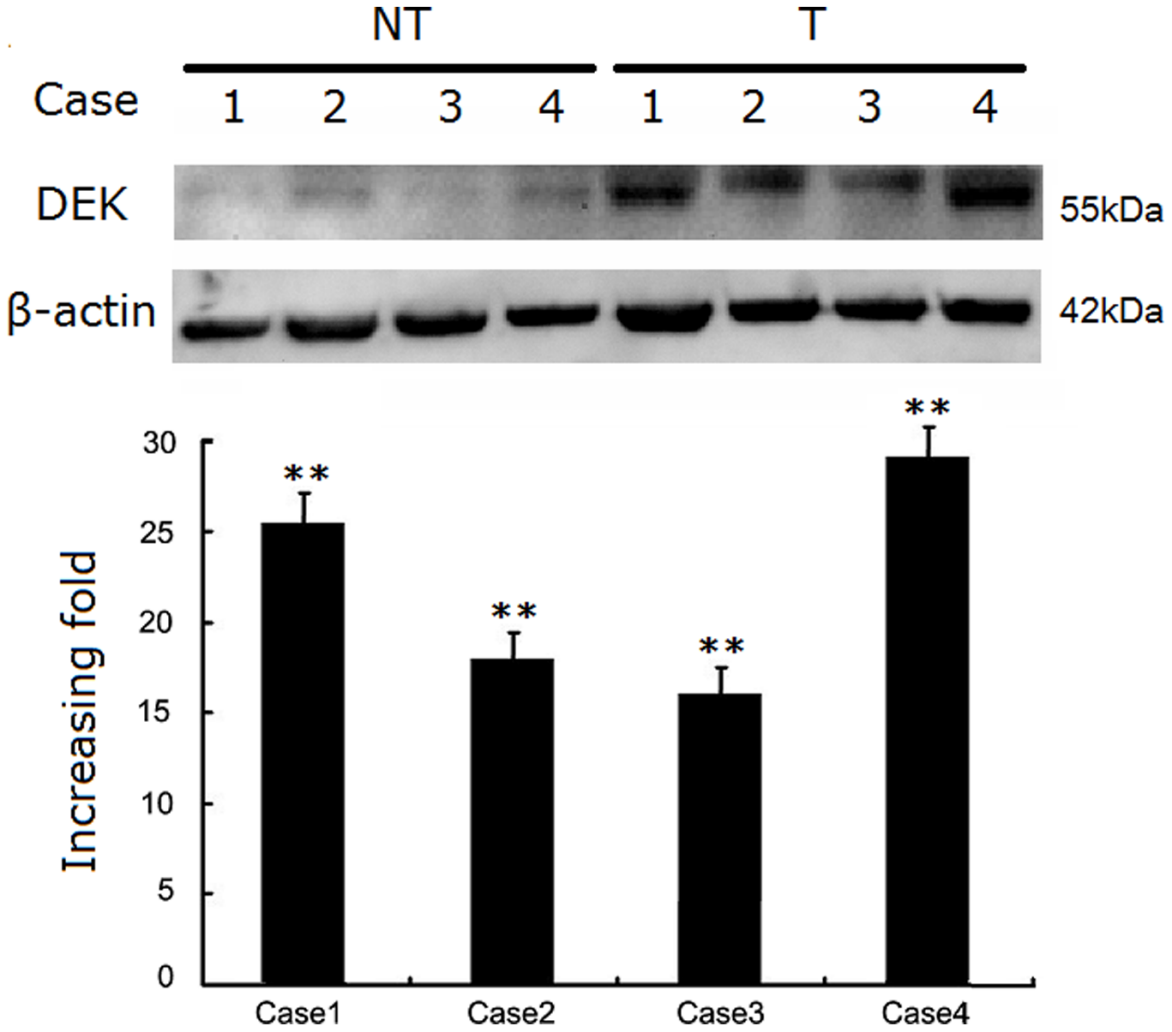
Western blotting analyses of the DEK protein. (A) Images of western blots of DEK protein expression in four matched pairs of CRC (T) and adjacent non-tumor tissues (NT). (B) Relative T/NT ratios of DEK protein expression levels in paired CRC and adjacent non-tumor tissues are shown (increased fold change, **P<0.01).

To demonstrate the role of DEK on CRC, we measured DEK protein levels in four cases of CRC with matched adjacent non-tumor fresh tissues. Western blot data showed robust DEK protein expression in CRC tissues compared with the matched adjacent non-tumor tissues (Fig. 1).

**Figure 2:**
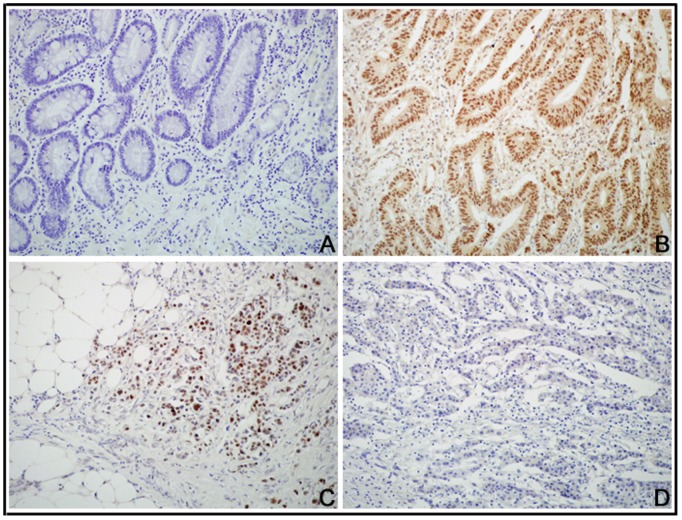
DEK protein expressed in CRC using IHC. (A) DEK protein staining was negative in adjacent normal colon tissues. (B) DEK protein showed diffusely and strongly nuclear positive staining in late-stage CRC. (C) DEK protein is positive in invasive cancer cells in the serosa of the colon. (D) DEK protein is negative in CRC without metastasis.

DEK protein expression showed a nuclear IHC staining pattern in CRCs (Fig. 2). The number of cells containing DEK protein (positive rate) was significantly higher in CRC tissues (80.0%, 44/55) than in normal adjacent mucosa (36.4%, 8/22) and in colorectal adenomas (16.7%, 3/18). Similarly, the strongly positive rate of DEK protein was 50.9% (28/55) in CRCs, which was significantly higher than in adjacent normal colon mucosa (18.2%, 4/22) and in colorectal adenomas (16.7%, 3/18) (Both *P*<0.001) (Table 2).

**Table 2:** DEK protein expression in colorectal adenocarcinoma.

Diagnosis	No. of cases	DEK expression				Positive rate (%)	Strongly positive rate (%)
		−	+	++	+++		
Normal	22	14	4	3	1	36.4%	18.2%
Adenoma	18	12	3	1	2	33.3%	16.7%
Carcinoma	55	11	16	16	12	80.0%**	50.9%**

**P<0.0001, compared with peripheral normal colorectal mucosa of cancers and adenomas. **Carcinoma:** colorectal adenocarcinoma; **Positive rate:** percentage of positive cases with +, ++, and+++staining score; **Strongly positive rate:** percentage of positive cases with++and+++staining score.

### Correlations between DEK expression and the Ki-67 index and apoptosis index in human CRC tissues

**Figure 3:**
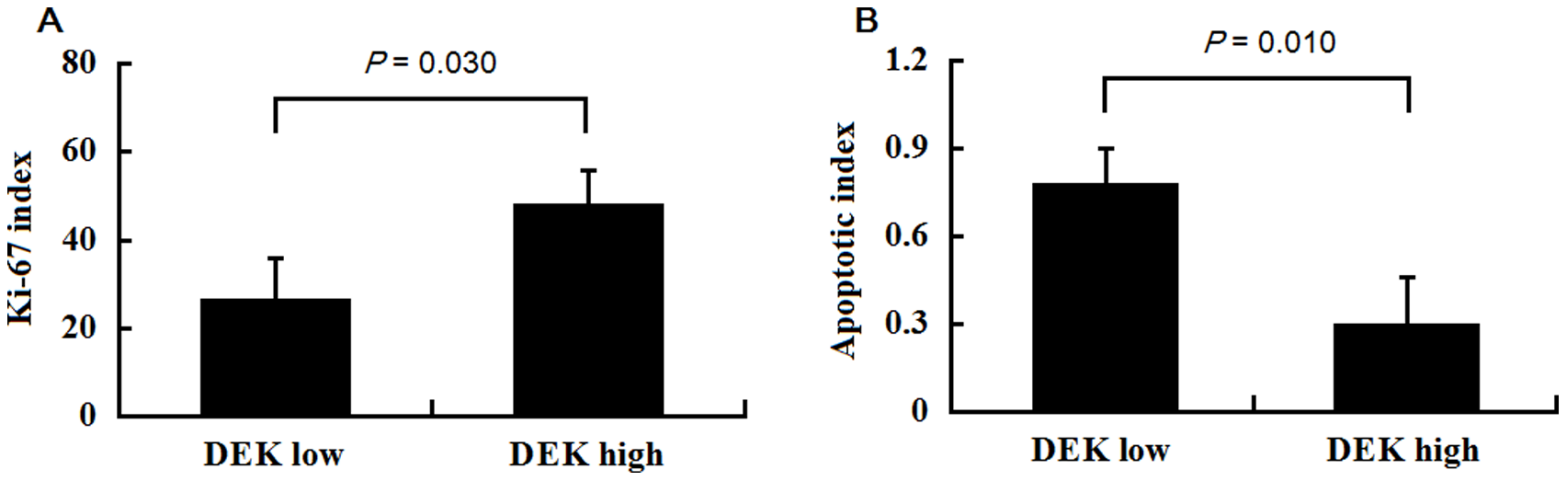
Correlations between DEK expression and the index of Ki-67 and apoptosis in CRC tissues. (A) Significant differences were observed in correlating DEK expression and the Ki-67 index (*P* = 0.030). (B) There was also a significant correlation between DEK expression and apoptotic index (*P* = 0.010). (DEK low, “−” and “+”; DEK high, “++” and “+++”. ).

We evaluated the correlations between DEK expression and the Ki-67 and apoptotic indices in human CRC tissues. The Ki-67 index was 26.60±8.12% in tissues with low DEK expression levels and 48.17±7.87% in tissues with high DEK expression levels (Fig. 3A). DEK expression levels and the Ki-67 index were positively correlated (*P* = 0.030). The apoptotic index was 0.78±0.10% in tissues with low DEK expression levels and 0.30±0.16% in tissues with high DEK expression levels (Fig. 3B). There was also a significant correlation between the DEK expression levels and the apoptotic index (*P* = 0.010).

### DEK expression in CRC cells by RT-PCR and western blot

**Figure 4:**
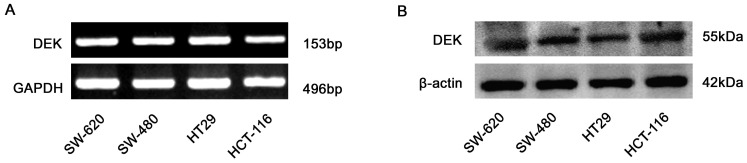
Expression levels of DEK were examined by RT-PCR (A) and western blot (B) in colorectal cancer cell lines, respectively.

The IHC data shown above and our previously reported data [5] suggest that DEK might be involved in the progression of CRCs and a good marker of proliferation and metastasis. Thus, we detected the expression levels of DEK mRNA and protein in several CRC cell lines, including SW-620, SW-480, HCT116, and HT29. RT-PCR data exhibited that all of the SW-620, SW-480, HT29 and HCT116 cells showed high expression levels of DEK mRNA (Fig. 4A). Similar results were observed for protein levels of these cell lines by western blot assays (Fig. 4B). Here we selected SW-620 cells, which have high proliferation and metastasis potential confirmed by previous reports [23], for the following experiments.

### Effects of DEK RNAi on cell proliferation of the colon cancer cell line, SW620

**Figure 5:**
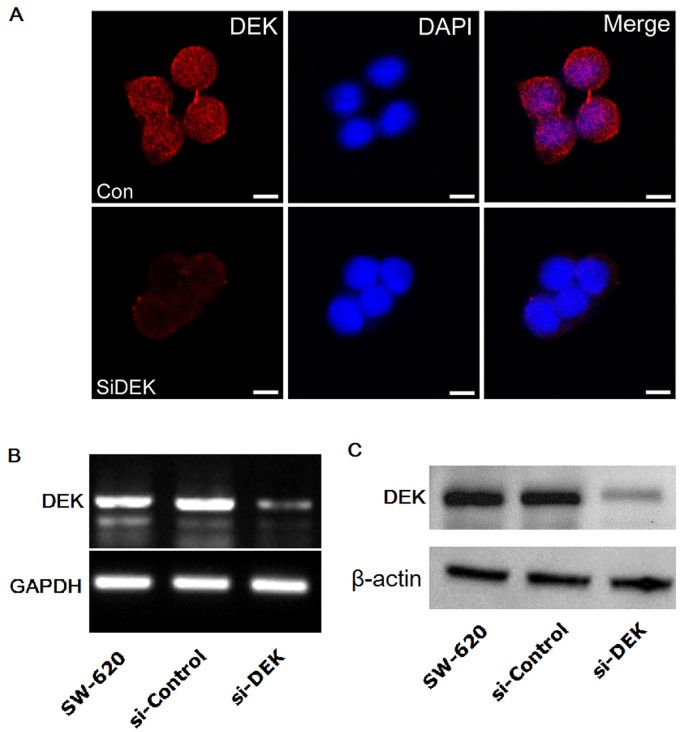
DEK gene expression in the human CRC cell line SW-620 after RNAi. (A) Nuclear localization of DEK protein is observed in siControl and siDEK SW-620 cells. DEK (red) localized to the SW-620 cell nuclei by immunofluorescence staining and confocal microscopy observation. DAPI staining (blue) was included to visualize the nucleus. (B) DEK mRNA expression in the human CRC cell line SW-620 after siControl and siDEK. (C) DEK protein expression in the human CRC cell line SW-620 after siControl and siDEK.

We transfected SW-620 cells with DEK RNAi, and found that DEK RNAi effectively downregulated the mRNA and protein expression levels of DEK in SW-620 cells by immunofluorescent staining, RT-PCR, and western blot analyses (Fig. 5A–C).

**Figure 6:**
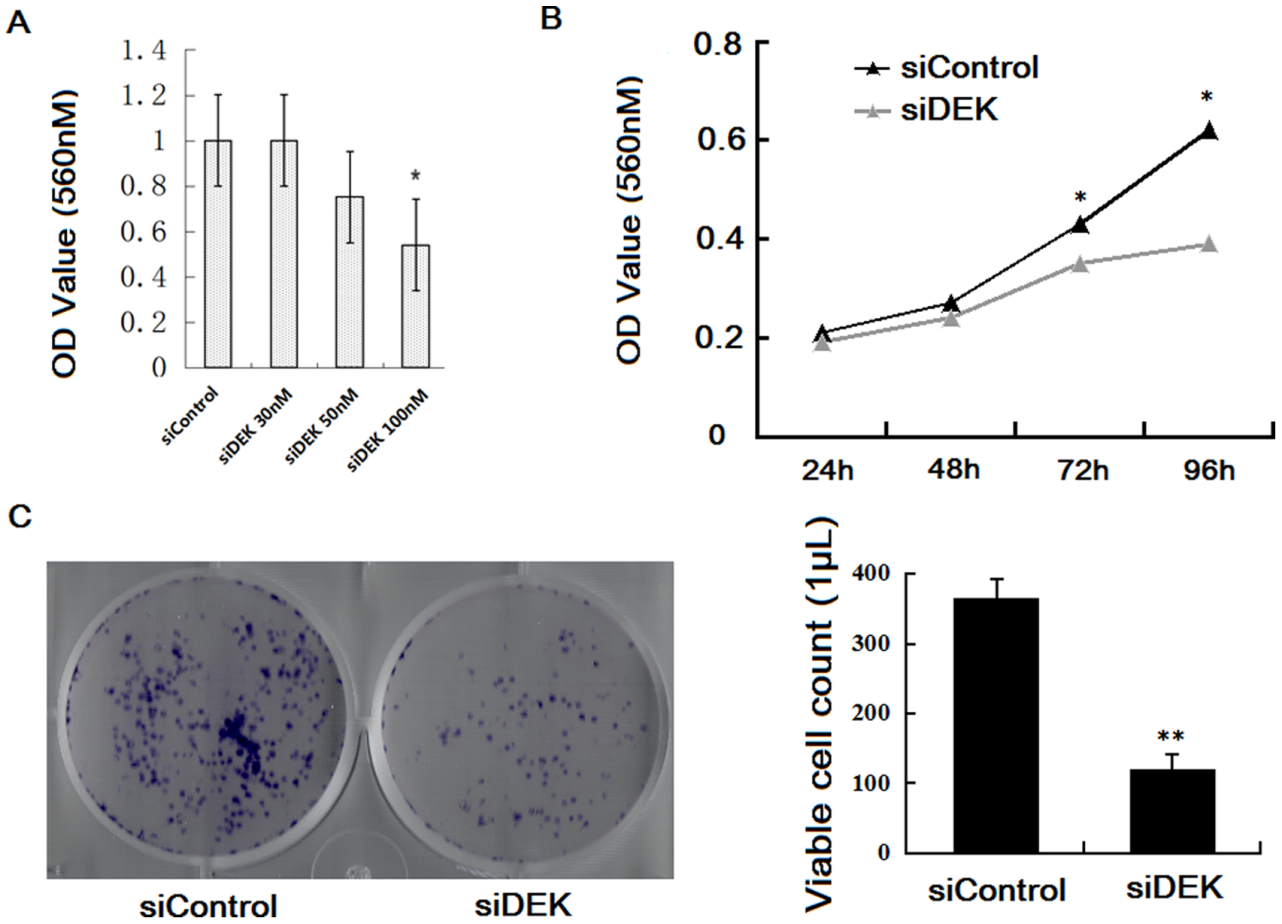
DEK contributes to the proliferation of the colorectal cancer cell line, SW-620. (A) MTT assay showed the siDEK (100 nM) mimics significantly reduced the proliferation of SW-620 cells relative to the siControl group (*P* = 0.034). (B) MTT assay after 24 h, 48 h, 72 h and 96 h for siControl and siDEK-transfected SW-620 cells, *P<0.05, respectively. (C) Colony formation assay showed siDEK treatment inhibited colony formation in SW-620 cells by about 70% (*P*<0.001).

The effects of DEK protein expression on cell growth were tested using an MTT assay. The growth rate of the 100 nM siDEK-treated cells decreased in a dosage-dependent manner (Fig. 6A). The absorbance values of the MTT assay after 72 h and 96 h were 0.43±0.02 and 0.62±0.03 for the siControl, and 0.35±0.03 and 0.39±0.02 for the siDEK-transfected SW-620 cells, both *P*<0.05, respectively (Fig. 6B). In addition, a colony formation assay showed SW-620 cells were efficiently reduced in the presence of siDEK (100 nM). In comparison, siDEK inhibited colony formation in SW-620 cells by about 70% (*P*<0.05) (Fig. 6C). These data indicated that there was a significant reduction in cell growth in the siDEK-transfected cells compared with the siControl cells.

### Effects of DEK expression on the apoptosis of SW-620 cells

**Figure 7:**
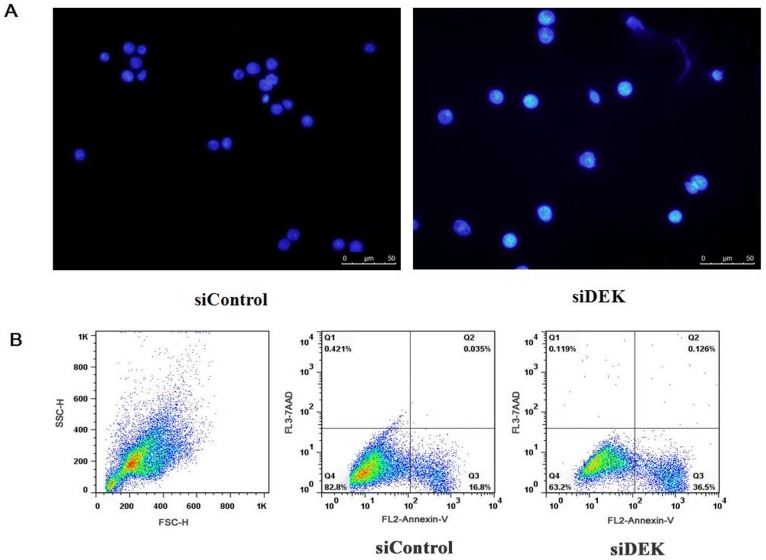
Knocking down DEK exacerbated the apoptosis of SW-620 cells. (A) Hoechst33342 staining showed that knockdown of the DEK gene induced apoptosis. (B) Transfected siDEK for 48 h markedly increased early-stage apoptotic cells indicated by a higher percentages of Annexin V-FITC+/7AAD- cells compared with the siControl group.

As shown in Fig. 7A, after SW-620 cells were transfected with 100 nM siDEK or siControl, the apoptotic rate was significantly increased in siDEK SW-620 cells compared with siControl-transfected SW-620 cells. The percentage of Annexin V-FITC+/7AAD- cells was 13.13% in the siDEK group, and 4.99% in the siControl group, indicating that DEK depletion increased the early apoptosis of SW-620 cells (Fig. 7B).

### Expression of p53/MDM2, caspase family, and Bcl-2/Bax proteins in siDEK-transfected cells

**Figure 8:**
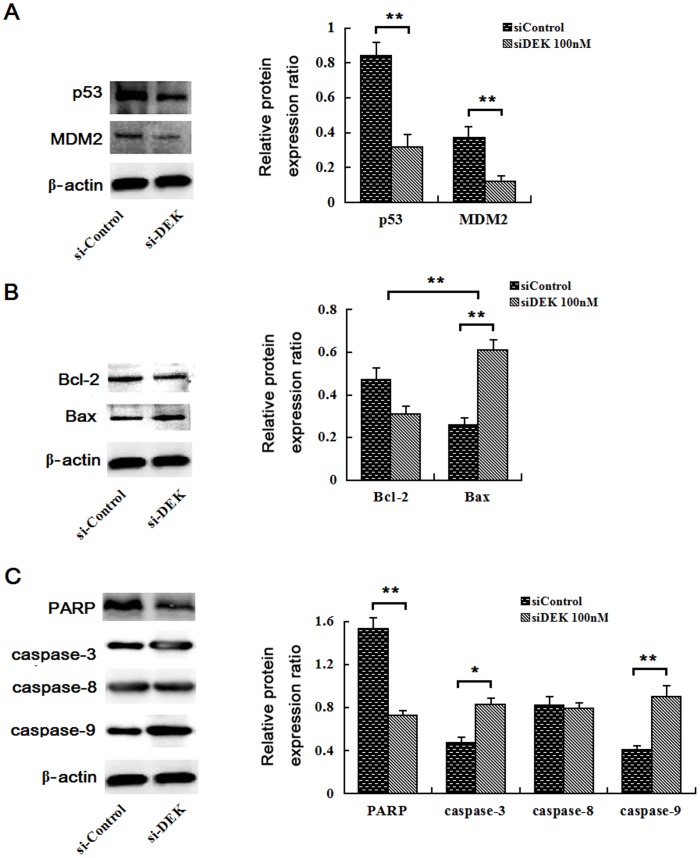
Expression of a number of factors related to proliferation and apoptosis in siDEK-transfected SW-620 cells. (A) Mutant-p53 and MDM2 proteins were significantly downregulated in siDEK-transfected SW-620 cells. (B) The ratio of Bcl-2/Bax was significantly reduced in siDEK-transfected SW-620 cells. (C) PARP protein expression was much higher in siDEK cells, and caspase-3 and −9 proteins were significantly lower in siDEK-transfected SW-620 cells than those in the siControl group. Caspase-8 protein levels remained unchanged.

As shown in Fig. 8A, the expression levels of mutant-p53 and MDM2 expression in siDEK-treated SW-620 cells decreased greatly in western blot assays, suggesting that DEK stimulates the growth of SW-620 cells through the p53/MDM2 pathway. Moreover, the expression of Bcl-2 was downregulated following siDEK treatment. In contrast, Bax expression was upregulated under the same conditions. These observations suggested that the Bcl-2/Bax pathway plays an important role in SW-620 cells’ progression that is regulated by DEK (Fig. 8B).

Additionally, our data also showed that cleaved caspase-3 and cleaved caspase-9 were decreased in the siDEK cells, while the expression level of caspase-8 did not change. However, cleaved poly ADP ribose polymerase (PARP) expression levels increased with siDEK application. These data suggested that caspase-3 and caspase-9, but not caspase-8, are involved in SW-620 cell apoptosis after siDEK transfection, indicating that siDEK also activated the caspase-dependent pathways (Fig. 8C).

The mechanism of DEK function in CRC was also confirmed in another CRC cell line HCT116, and found the similar results with that in SW620 cells. The data was provided as the supplemented figures (Fig. S1–S3).

## Discussion

DEK gene amplification and upregulated expression have been described in multiple cancer types including hepatocellular carcinoma, bladder cancer, and melanoma [7], [16], [24]. The works of Khodadoust et al [7] showed that DEK expression levels can distinguish benign nevi from malignant melanomas, indicating that this protein may prove highly useful for differential diagnoses. Recently, Datta et al. [24] reported that the oncoprotein DEK was upregulated in bladder cancer tissues in comparison with normal counterparts as determined by western blots. Indeed, DEK protein was present in the voided urine of patients with low- and high-grade bladder cancers, suggesting that DEK could be used as a biomarker for detection of bladder cancer using patient urine samples. Our current study is the first to report on the oncogenic activities of DEK in CRC by depleting DEK, and to define functional and molecular mechanisms for DEK in CRC pathogenesis.

Our previous study [5] showed that the strongly positive rate of DEK protein was 48.62% (53/109) in colorectal cancers, which was significantly higher than that in either adjacent normal colon mucosa (9.17%, 10/109) or colorectal adenomas (13.46%, 7/52). DEK overexpression in CRCs was positively correlated with tumor size, grade, lymph node metastasis, serosal invasion, late stage, and disease-free survival and 5-year survival rates. Further analyses showed that patients with late-stage CRC and high DEK expression had worse survival rates than those with low DEK expression. Moreover, multivariate analysis showed high DEK expression, serosal invasion, and late stage are significant independent risk factors for mortality in CRC. In this work, western blot and IHC staining analyses showed that high DEK expression was significantly more common in CRC tissues than in either adjacent normal colorectal mucosa or colorectal adenomas.

The Ki-67 antigen is expressed by proliferating cells during the G1, S, G2, and M phases, but not during the G0 phase (resting cells) [25]. Ki-67 is used as a marker of tumor proliferation and aggressiveness, and it can have a major effect on the prognosis of patients with CRC [26]–[27]. Our previous results demonstrated that the DEK protein expression pattern was very similar to the Ki-67 antigen as a proliferating marker in uterine cervical cancers [28]. A positive correlation between DEK expression and the Ki-67 or apoptotic index in CRC tissues was also found in this study.

Wise-Draper et al. [29] employed RNAi approaches for the specific targeting of DEK in cancer and primary cells, and found that DEK depletion in HeLa cervical cancer cells resulted in the induction of apoptosis. Similar results were obtained with primary human keratinocytes, implicating DEK as a cancer and primary cell survival factor. In this study, we also detected the association between DEK overexpression and more malignant behaviors of CRC using SW-620 and HCT116 human CRC cells with and without RNAi treatment. The cell apoptosis analysis revealed that DEK depletion increased the early apoptosis of SW-620 and HCT116 cells. Despite this, we also observed that DEK depletion inhibited cell growth in an MTT assay and colony formation assay. These results agreed with the results of the Ki-67 and apoptosis index in CRC tissue sample. Our findings indicate that DEK regulates CRC cell growth and survival *in vitro*.

The human p53 protein is the most frequently inactivated tumor suppressor gene in human cancer and is involved in the control of cellular proliferation in response to stress [30]. However, p53 has a short half-life and is maintained at low or undetectable levels by continual proteolytic degradation in normal cells. Sustained degradation of p53 is mediated mainly by the E3 ligase MDM2 [31]. By binding p53, MDM2 blocks the transactivation domain of p53 and therefore inhibits its transcriptional activity [32]. Challenged with stress signals such as hypoxia or DNA damage, the feedback loop of MDM2-p53 is disturbed, leading to apoptosis or malignancy [33]. Khodadoust’s study suggested a novel role for DEK in cell survival, involving the destabilization of p53 in a manner that is likely to contribute to human carcinogenesis [7]. In addition, Wise-Draper et al. also considered that cell death in response to DEK depletion was accompanied by increased protein stability and transcriptional activity of the p53 tumor suppressor, and subsequent upregulation of known p53 target genes such as Bax [29]. Bax, a pro-apoptotic factor of the Bcl-2 family, is located in a monomeric form in the cytosol or loosely attached to the membranes under normal conditions. Following a death stimulus, cytosolic and monomeric Bax translocate to the mitochondria, where they become integral membrane proteins. In the mitochondrial membrane, Bax proteins cross-link as homodimers, allowing for the release of factors from the mitochondria to propagate the apoptotic pathway [34]–[38]. Pro-apoptotic proteins promote the release of cytochrome c from the mitochondria, initiating the apoptotic cascade. Cytochrome c activates caspase-9, which cleaves and activates downstream effector proteases, such as caspase-3, leading to apoptosis [39]. Once activated, caspase-3 cleaves PARP into two fragments, promoting DNA fragmentation and triggering apoptosis [40]. In this study, our data showed that down-expression of DEK by transfection of siDEK resulted in greatly reduced mutant-p53 and MDM2 expression in both SW-620 and HCT116 cells, suggesting that DEK inhibits apoptosis of CRC cells via the p53 pathway. In addition, expression of the pro-apoptotic Bax protein increased and Bcl-2 protein levels decreased in both SW-620 and HCT116 siDEK cells. Our data agree with Xiao et al's report [41] and suggest the ratio of Bcl-2/Bax is important for the survival of drug-induced apoptosis in cancer cells, rather than the expression level of Bcl-2 alone. Moreover, caspase-3 and caspase-9 protein levels significantly increased in the SW-620 and HCT116 siDEK cells, and PARP protein levels significantly decreased in this study. These data propose that caspase-3 and caspase-9 are involved in SW-620 and HCT116 cell apoptosis after siDEK transfection, indicating that the caspase-dependent pathways could be inhibited by DEK overexpression.

**Figure 9:**
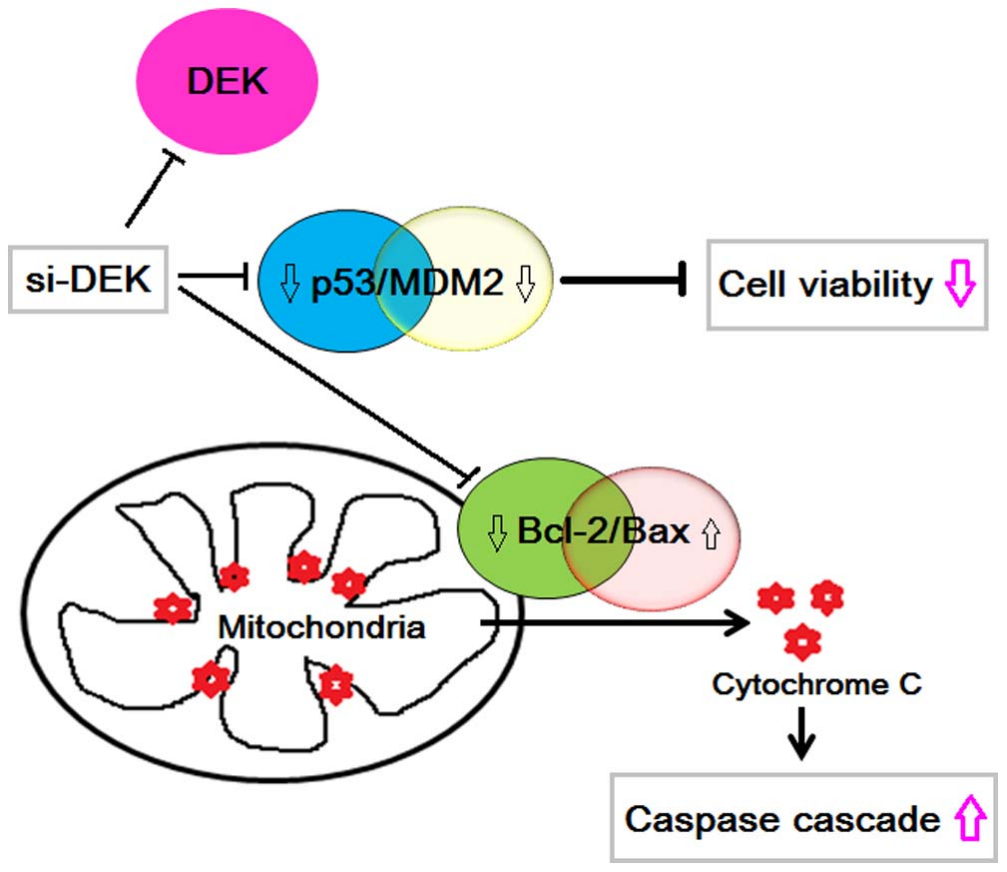
The mechanistic diagram to schematically illustrate the targets of DEK apoptotic action in CRC cells. DEK depletion was accompanied by decreased mutant-p53 and pro-apoptotic protein Bax, which promotes the release of cytochrome C from the mitochondria.

Numerous studies using DEK specific RNAi have shown a significant increase in cellular apoptosis or senescence through p53-dependent and -independent mechanisms [42]. Relevant mechanisms include p53 stabilization, down-regulation of histone H3 Lys9 and H4 Lys5 overall acetylation level, induction of paracrine Wnt signaling, interacting with AP-2α, and the transcriptional repression of myeloid cell leukemia 1 (MCL-1) and hTERT [43]–[44]. Additionally, there are several DEK related proteins, such as Annexins, Enolase 1, Lamin A and Glutathione-S-transferase omega 1, etc., are correlated with the cell apoptosis [45]. The present work demonstrated that DEK depletion was accompanied by increased protein stability and transcriptional activity of the p53 tumor suppressor, and subsequent upregulation of pro-apoptotic proteins, such as Bax, which promotes the release of cytochrome c from the mitochondria (caspase-3 and −9 increased), initiating the apoptotic cascade (Fig. 9). How these various programs are selectively altered is a topic of ongoing studies.

## Supporting Information

Figure S1 **DEK protein expression in the human CRC cell line HCT116 after siControl and siDEK.**
(TIF)

Figure S2 **Knocking down DEK exacerbated the apoptosis of HCT116 cells.** (A) Hoechst33342 staining showed that knockdown of the DEK gene induced apoptosis. (B) Transfected siDEK for 48 h markedly increased early-stage apoptotic cells indicated by a higher percentages of Annexin V-FITC+/7AAD- cells compared with the siControl group.
(TIF)

Figure S3 **Expression of a number of factors related to proliferation and apoptosis in siDEK-transfected HCT116 cells.** (A) Mutant-p53 and MDM2 proteins were significantly downregulated in siDEK-transfected HCT116 cells, and the ratio of Bcl-2/Bax was significantly reduced in siDEK-transfected HCT116 cells. (B) PARP protein expression was much higher in siDEK cells, and caspase-3 and −9 proteins were significantly lower in siDEK-transfected HCT116 cells than those in the siControl group. Caspase-8 protein levels remained unchanged.
(TIF)

Table S1 **List of primers of siDEK.** Three different RNAi (siDEK1, siDEK2, and siDEK3) were designed to deplete DEK gene from RiboBio, Guangzhou, China. The results showed that both siDEK1 and siDEK2 could effectively knockdown the DEK expression compared with the siControl transfected group, however, siDEK3 has no any effect for DEK knockdown. So, we selected siDEK1 for interfering and targeting to the DEK gene in this study.
(DOC)
