# Supplementary figures and images for: Mechanisms Underlying Cancer Growth and Apoptosis by DEK Overexpression in Colorectal Cancer
Source: PLoS One. 2014 Oct 23;9(10):e111260. doi: 10.1371/journal.pone.0111260 (PMC4207817; doi:10.1371/journal.pone.0111260)

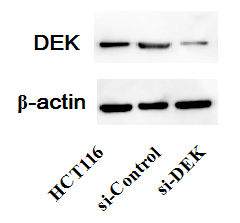

Supplement: Figure S1 — DEK protein expression in the human CRC cell line HCT116 after siControl and siDEK. (TIF) [file pone.0111260.s001.tif]

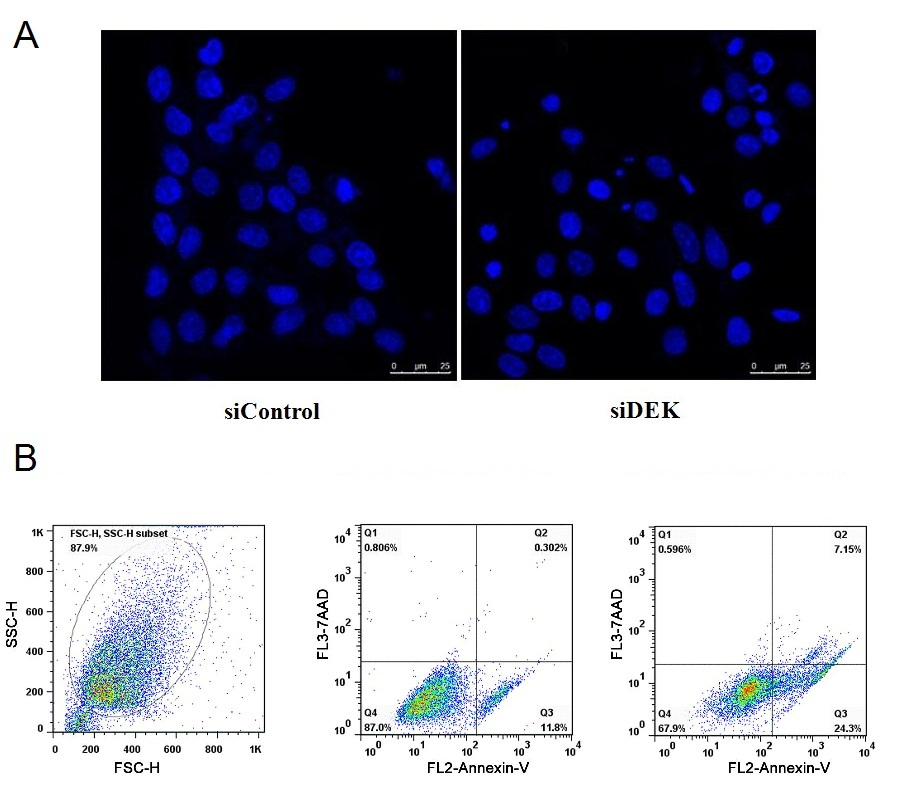

Supplement: Figure S2 — Knocking down DEK exacerbated the apoptosis of HCT116 cells. (A) Hoechst33342 staining showed that knockdown of the DEK gene induced apoptosis. (B) Transfected siDEK for 48 h markedly increased early-stage apoptotic cells indicated by a higher percentages of Annexin V-FITC+/7AAD- cells compared with the siControl group. (TIF) [file pone.0111260.s002.tif]

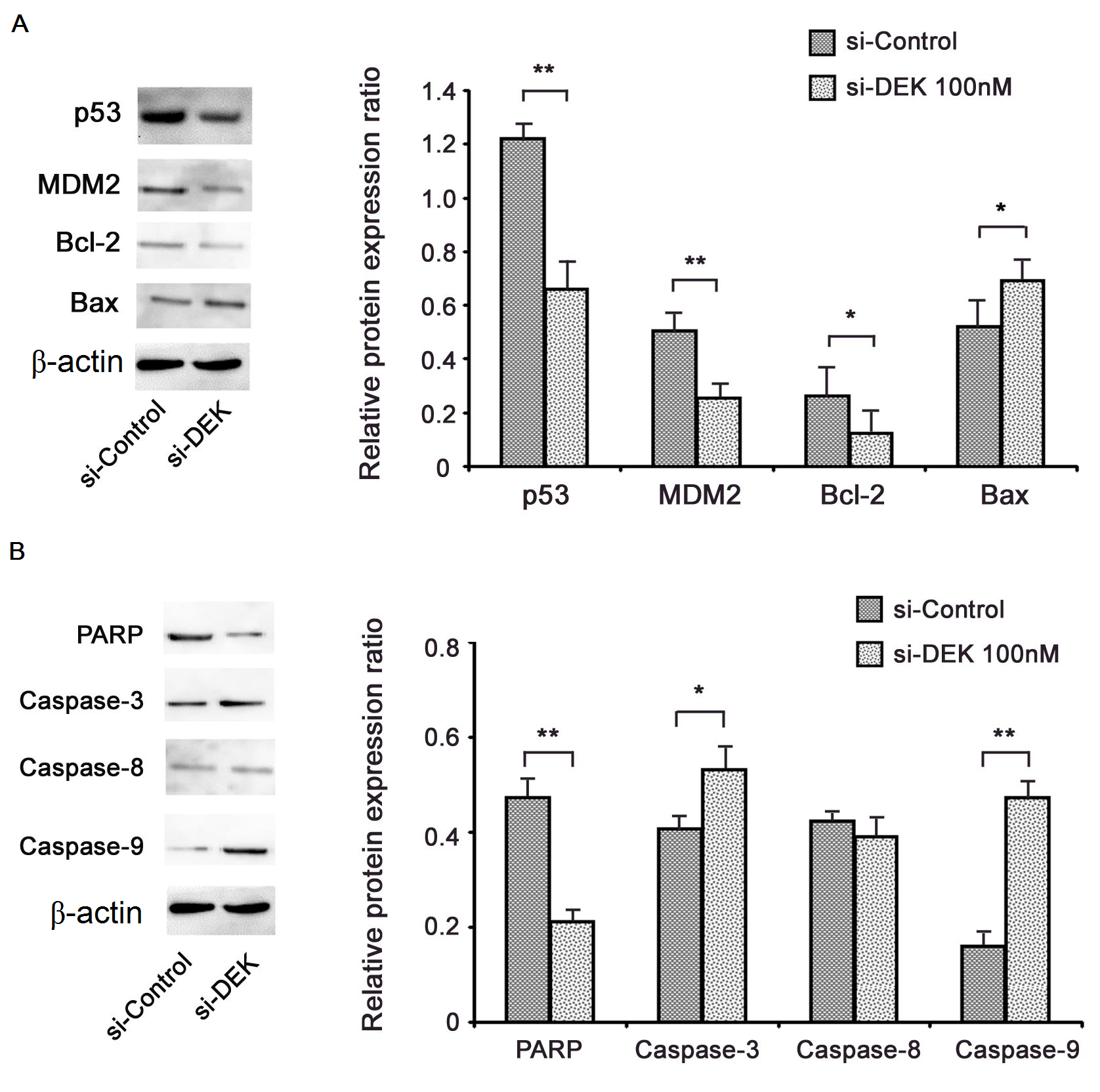

Supplement: Figure S3 — Expression of a number of factors related to proliferation and apoptosis in siDEK-transfected HCT116 cells. (A) Mutant-p53 and MDM2 proteins were significantly downregulated in siDEK-transfected HCT116 cells, and the ratio of Bcl-2/Bax was significantly reduced in siDEK-transfected HCT116 cells. (B) PARP protein expression was much higher in siDEK cells, and caspase-3 and −9 proteins were significantly lower in siDEK-transfected HCT116 cells than those in the siControl group. Caspase-8 protein levels remained unchanged. (TIF) [file pone.0111260.s003.tif]
