# Supplementary material for: Mechanisms Underlying Cancer Growth and Apoptosis by DEK Overexpression in Colorectal Cancer
Source: PLoS One. 2014 Oct 23;9(10):e111260. doi: 10.1371/journal.pone.0111260 (PMC4207817; doi:10.1371/journal.pone.0111260)
Supplement: Table S1 — List of primers of siDEK. Three different RNAi (siDEK1, siDEK2, and siDEK3) were designed to deplete DEK gene from RiboBio, Guangzhou, China. The results showed that both siDEK1 and siDEK2 could effectively knockdown the DEK expression compared with the siControl transfected group, however, siDEK3 has no any effect for DEK knockdown. So, we selected siDEK1 for interfering and targeting to the DEK gene in this study. (DOC) [file pone.0111260.s004.doc]

**Table S1.** List of primers used in this study

| SiDEK1 | sense | 5‘ CGAACCAAAUGUCCUGAAA dTdT 3‘ |
| --- | --- | --- |
|  | antisense | 3‘ dTdT GCUUGGUUUACAGGACUUU 5‘ |
| SiDEK2 | sense | 5‘ CAAUGAAACAGAUUUGCAA dTdT 3‘ |
|  | antisense | 3‘ dTdT GUUACUUUGUCUAAACGUU 5‘ |
| SiDEK3 | sense | 5‘ GAAAGUGAGUCUGAGGAUA dTdT 3‘ |
|  | antisense | 3‘ dTdT CUUUCACUCAGACUCCUAU 5‘ |
